# Enhanced positronium lifetime imaging through two-component reconstruction in time-of-flight positron emission tomography

**DOI:** 10.3389/fphy.2024.1429344

**Published:** 2024-07-14

**Authors:** Zhuo Chen, Chien-Min Kao, Hsiun-Hsiung Huang, Lingling An

**Affiliations:** 1 Graduate Interdisciplinary Program in Statistics and Data Science, University of Arizona, Tucson, AZ, United States; 2 Department of Radiology, University of Chicago, Chicago, IL, United States; 3 Department of Statistics and Data Science, University of Central Florida, Orlando, FL, United States; 4 Department of Biosystems Engineering, University of Arizona, Tucson, AZ, United States

**Keywords:** positronium lifetime imaging, image reconstruction, time-of-flight positron emission tomography, two-component model, maximum likelihood estimation

## Abstract

Positronium lifetime imaging (PLI) is a newly demonstrated technique possible with time-of-flight (TOF) positron emission tomography (PET), capable of producing an image reflecting the lifetime of the positron, more precisely ortho-positronium (o-Ps), before annihilation, in addition to the traditional uptake image of the PET tracer. Due to the limited time resolution of TOF-PET systems and the added complexities in physics and statistics, lifetime image reconstruction presents a challenge. Recently, we described a maximum-likelihood approach for PLI by considering only o-Ps. In real-world scenarios, other populations of positrons that exhibit different lifetimes also exist. This paper introduces a novel two-component model aimed at enhancing the accuracy of o-Ps lifetime images. Through simulation studies, we compare this new model with the existing single-component model and demonstrate its superior performance in accurately capturing complex lifetime distributions.

## 1 Introduction

Positron emission tomography (PET) is a major medical imaging modality that has been routinely used for diagnosing diseases such as cancer and Alzheimer’s disease [1]. In PET, one administrates molecules labeled with a positron-emitting isotope that targets certain signatures associated with these diseases so that the presence and/or the grade of disease can be manifested by the tissue uptake of the molecules at an appropriate time post-administration. PET imaging is based on coincidence detection of gamma-ray photons that are created when a positron released by the isotope annihilates with an electron. The released positrons do not annihilate instantly, and until recently, little attention has been paid to the processes leading to annihilation. In tissues, when sufficiently slowing down after traveling a certain distance (known as the positron range that is characteristic of the isotope), a positron can annihilate directly with an electron. As an alternative to such direct annihilation (DA), some positrons may form a short-lived meta-stable particle called positronium. There are two types of positronium: para-positronium (p-Ps) and ortho-positronium (p-Ps). The duration for which a positron exists before annihilation *τ* can be described probabilistically by an exponential distribution, $p(\tau )=\lambda e^{-\lambda \tau }$, τ≥0 so that the mean duration of existence, called lifetime, equals 1/λ. The three populations described above have different rate constants, with those of the DA and p-Ps populations substantially larger than that of o-Ps (or the DA and p-Ps populations have substantially shorter lifetimes than o-Ps). Significantly, the rate constant of o-Ps is sensitive to the local tissue properties surrounding o-Ps. The decay constant for DA and p-Ps also changes in the medium. However, the changes in DA and p-Ps decay constants in a medium are much less pronounced than the change in the o-Ps decay rate [2]. Positronium lifetime imaging (PLI) aims to exploit this feature, producing rate-constant image (or its inverse, the lifetime image) of o-Ps for providing diagnostic information for disease in addition to that given by the uptake of PET molecules. Readers are referred to Moskal et al. [3] for in-depth discussion of the physics underlying PLI.

**FIGURE 1:**
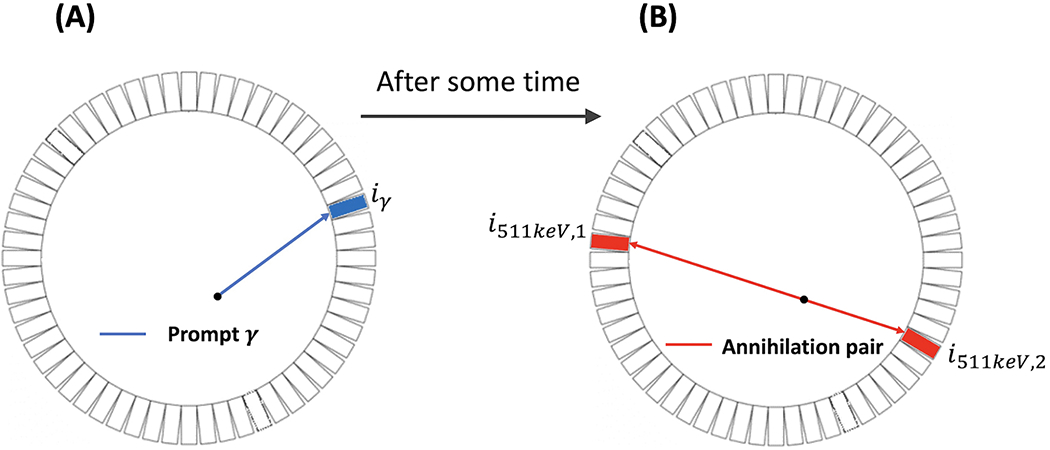
Two-dimensional illustration of the emission and detection of prompt ***γ*** (blue) and the annihilation pair (red). **(A)**: emission of the prompt *γ* ray. **(B)**: emission of the annihilation pair (two photons with an energy of 511 keV) each in opposite directions.

As illustrated in Figure 1B, annihilation of a positron releases two gamma-ray photons with an energy of 511 keV that travel in opposite directions [4]. Based on this, PET detectors capture two 511-keV photons in coincidence to define a line of response (LOR) along which the annihilation is expected to take place. PET imaging describes the distribution of PET molecules in the body, and the resulting image is commonly referred to as an activity image [5]. In conventional PET, the detection time of the gamma rays is used only for determining the coincidence of two photons and the LOR. It does not determine which location on the LOR is the origin of the two photons, and hence of the positron if the positron range is negligible [6]. In time-of-flight (TOF) PET, the accuracy in time measurement is improved such that a certain degree of localization of the positron on the LOR is based on the difference in the detection time of two photons in coincidence. In the standard practice, an LOR is divided into multiple segments, which are often called a line of segment (LOS), and events detected at the LOR are further assigned to these segments according to their TOF values. The imaging model now relates the radioactivity distribution to the number of events detected at all the LOS of the system. TOF-PET is beneficial because it can increase the signal-to-noise ratio (SNR) of the resulting activity image. Better TOF resolution, typically reported using the coincidence resolving time (CRT) in picosecond (ps), leads to a better image SNR. Improving the CRT has been a hot topic for research. In the past decades, the CRT of the clinical TOF-PET system has been improved from approximately 600 ps to slightly better than 200 ps [7].

Recently, PLI was introduced [8–10] and *ex vivo* and *in vivo* positronium lifetime images were demonstrated using a novel multi-photon J-PET system [11, 12]. Contrary to the traditional PET/TOF-PET, PLI employs a non-pure positron-emitter, such as Sc-44, that emits a positron and a prompt gamma ray essentially at the same time and detects three-photon coincidences that include the prompt gamma and the two 511-keV annihilation photons, in addition to standard two-photon coincidences [13, 14]. In three-photon events, the detection time of the prompt gamma (depicted in blue in Figure 1A) can provide the “start” reference for measuring the elapse time of the positron before annihilation (depicted in red in Figure 1B) [2, 15]. As described above, the lifetime distribution of positrons contains three populations, and the interest in PLI is to obtain the lifetime image of the o-Ps population. In this paper, we present a two-component model that includes a slow-decay component representing the o-Ps population and a fast-decay component that represents the DA and p-Ps populations combined. The lifetime of o-Ps is very sensitive to the size of the voids (free volume between the atoms) and the concentration of molecules such as oxygen molecules [16]. There are pieces of evidence that o-Ps may provide information about disease progression in an initial stage [17, 18]. It has been shown in recent studies that the o-Ps lifetime in healthy adipose tissue differs from the o-Ps lifetime in cardiac myxoma tumors [19].

The FWHM in centimeter (cm) of the localization uncertainty illustrated in Figure 1 equals 0.015 × CRT when the CRT is expressed in ps. The best CRT of the state-of-the-art TOF-PET systems is approximately 200 ps [20, 21], corresponding to a localization uncertainty of 3 cm. Therefore, an LOS sees a mixture of events in a substantial region within which the o-Ps lifetime generally varies. Prior research studies on PLI by Qi and Huang [22]; Huang et al. [23]; Chen et al. [24]; and Shopa and Dulski [25, 26] have presented probabilistic formulations that describe the PLI data acquired by TOF-PET systems having a finite CRT, allowing accurate reconstruction of the rate-constant image. These works predominantly focused on the lifetime of o-Ps and exclusively modeled only o-Ps decays in the simulated data. In this paper, we consider more realistic simulation data by including all three positron populations.

## 2 Methods

### 2.1 Data simulation

PLI events of the o-Ps, DA, and p-Ps populations were simulated according to their respective population weights and rate-constant images by using the Monte Carlo methods and scanner configurations previously described in Huang et al. [23]. Briefly, we considered two-dimensional (2D) imaging and only triple-coincidence events. The scanner contained 364 identical detectors on a diameter of 57.2 cm. The activity image was first scaled so that its summed intensity equaled the desired number of events to produce. The number of decay in an image pixel was then drawn from a Poisson distribution whose mean equaled the scaled pixel intensity. For each decay, the prompt gamma and annihilation photons were simulated to emit in two random angles independently drawn from a uniform distribution over [0, 2*π*). The detectors seeing these radiations were then determined accordingly. As discussed above, the elapse time between the prompt gamma and annihilation photons was sampled from a three-component exponential mixture model using the population weights and rate constants specified for the pixel in which the decay was located. To account for uncertainty in time measurement, every detection time was perturbed by a random number drawn from a Gaussian distribution having zero mean and standard deviation (SD) *σ*_1_. For a specified coincidence resolving time (CRT) for the scanner, the TOF bin size was chosen to be half of the CRT and $\sigma_{1}=\sigma_{t}/\sqrt{2}$, where $\sigma_{t}=CRT/2.35$ was the SD value corresponding to the CRT. Attenuation, scattering, random events, and position range were not simulated.

**FIGURE 2:**
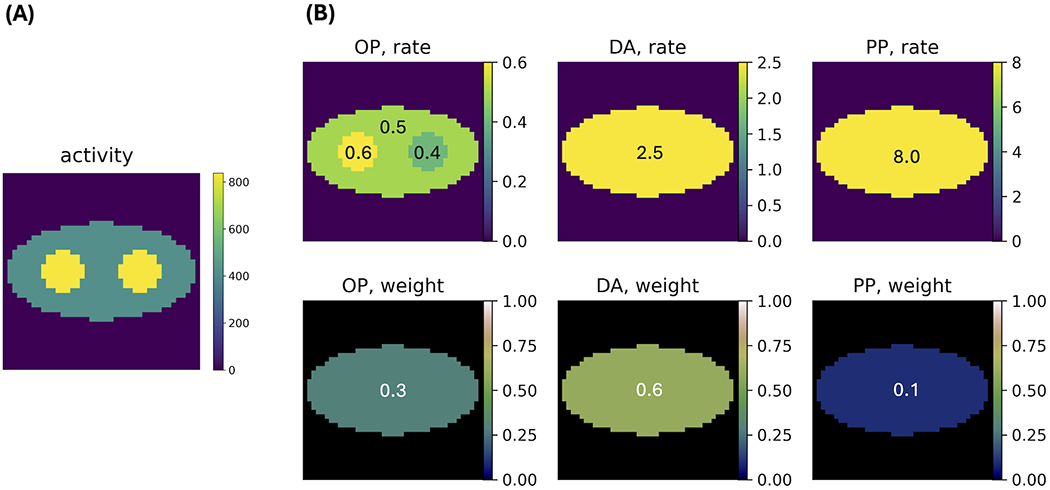
**(A)** Example of the ground truth of the activity image, with an average of 500 decays per pixel. **(B)** Ground truth of the rate-constant and population-weight images for o-Ps (OP), direct annihilation (DA), and p-Ps (PP).

PLI events of the three positron populations were generated using representative population weights (DA: p-Ps: o-Ps ≈ 0.6: 0.1: 0.3) and rate constants ($\lambda_{DA}\approx 2.5{ns}^{-1}$; $\lambda_{p-Ps}\approx 8.0{ns}^{-1}$; $\lambda_{o-Ps}\approx 0.5{ns}^{-1}$), as reported in the literature [10]. Figure 2 displays the ground truth of the activity image and rate-constant images and population-weight images for each component. The activity image contains two discs on an elliptical background. Pixels in the two discs have two times the activity in the elliptical background. The o-Ps rate-constant image consists of the same-sized discs and background ellipse. The two discs have different *λ* values (0.4 and 0.6 ns^−1^) from the background ellipse (0.5 ns^−1^). Outside the ellipse, since no annihilations occur, *λ* is not measured. Therefore, we arbitrarily set *λ* = 0 ns^−1^ for this region. As mentioned above, the DA and p-Ps rate constants are not sensitive to the environment. Therefore, we consider uniform rate-constant images for them; both consisting of only the background ellipse having *λ* values of 2.5 ns^−1^ (for DA) and 8.0 ns^−1^ (for p-Ps). Similarly, the population weights are not sensitive to the environment and therefore uniform population-weight images are used, with values of 0.3, 0.6, and 0.1 for o-Ps, DA, and p-Ps, respectively. The elliptical region where *λ* > 0 in the rate-constant images is referred to as the region of interest (ROI). All the images are discretized into square pixels of 3.27 × 3.27 mm^2^, with 41 × 41 pixels.

Since p-Ps has a low population weight and also decays substantially faster than others, its presence in the PLI data is not significant, and we stipulate that it is sufficient to consider a PLI model that includes two components, including a fast-decay component representing the DA population and a slow-decay component representing the o-Ps population. We evaluate the performance of using this new two-component model for reconstructing the o-Ps rate-constant image.

We implemented the proposed method using the combinations of the following simulation settings.

1. Activity image used for rate-constant image reconstruction:
  - true activity image (referred to as ***f***);
  - estimated activity image (referred to as $\overset{ˆ}{f}$).
2. Event size, calculated as the product of the number of pixels in the ROI (627 in the phantom used), and a multiplier of choice, which represents the average number of PLI events per pixel.
  - 627 × 100;
  - 627 × 200;
  - 627 × 500; and
  - 627 × 1,000;
3. TOF resolution expressed in SD *σ_t_*, equal to CRT/2.35
  - 0.085 ns, representing a 200-ps CRT, approximately the best TOF currently achievable
  - 0.16 ns, representing a 377-ps TOF PET system; and
  - 0.242 ns, representing a 570-ps TOF PET system.

For each simulation setting, we conducted 20 independent runs. The final reconstructed rate-constant images were derived by averaging the rate-constant images obtained from these 20 runs.

### 2.2 Two-component reconstruction model

As described by Huang et al. [23], when focusing exclusively on o-Ps decays, the elapse time measured for event *k*, $\tau_{k}$, is modeled using an exponentially modified Gaussian (EMG) distribution, which is the convolution of an exponential distribution describing the lifetime distribution with a Gaussian distribution describing the uncertainty in measurement of elapse time [3]. In the extended model that includes both slow- and fast-decay components, $\tau_{k}$ follows a mixture of two EMG distributions: one representing the o-Ps population and another the DA population. As mentioned, even though the p-Ps population is included in the simulated data, in this study, we exclude the p-Ps population from the reconstruction model due to their negligible presence in the data. Our results below will demonstrate that this simplification does not noticeably compromise reconstruction accuracy.

The density of $\tau_{k}$ is expressed as

(1) $$w_{1,j}\cdot EMG\left(\tau_{k};\lambda_{1,j},\sigma^{2}\right)+w_{2,j}\cdot EMG\left(\tau_{k};\lambda_{2,j},\sigma^{2}\right),$$

where $w_{1,j}+w_{2,j}=1$ and EMG (·) is the density of a EMG distribution and has the following form:

(2) $$\text{EMG}\left(\tau ;\lambda ,\sigma^{2}\right)=\frac{1}{2}\lambda e^{-\lambda \left(x-\frac{1}{2}\sigma^{2}\lambda \right)}\left(1+\text{erf}\left(\frac{\tau -\lambda \sigma^{2}}{\sqrt{2}\sigma }\right)\right),$$

where the value for σ is determined from the CRT, which equals $(\sqrt{3}/2)\sigma_{t}$. Let ${𝒲}_{N_{k}}=\left\{\left(c_{k},\tau_{k}\right):k=1,\ldots ,N_{k}\right\}$ denote the acquired list-mode containing *N_k_* events where *c_k_* and $\tau_{k}$ are the LOS and the measured elapse time for event *k*, respectively. It is shown in Huang et al. [23] that the log-likelihood function of ${𝒲}_{N_{k}}$ given the activity image ***f*** is

(3) $$\ell \left(\lambda_{1},w_{1};\lambda_{2},f,{𝒲}_{N_{k}}\right)\;\;=\overset{N_{k}}{\underset{k=1}{\sum }}\text{log}\left\{\overset{N_{j}}{\underset{j=1}{\sum }}H_{c_{k},j}f_{j}\left[w_{1,j}\cdot \text{EMG}\left(\tau_{k};\lambda_{1,j},\sigma^{2}\right)+w_{2,j}\cdot \text{EMG}\left(\tau_{k};\lambda_{2,j},\sigma^{2}\right)\right]\right\}.$$

Therefore, given an estimate for the activity image $\overset{ˆ}{f}$ and assuming that ***λ***_2_ is known, the maximum likelihood estimation (MLE) of the o-Ps rate-constant image ${\overset{ˆ}{\lambda }}_{1}$ and its weight ${\overset{ˆ}{w}}_{1}$ is obtained by

(4) $$\left({\overset{ˆ}{\lambda }}_{1},{\overset{ˆ}{w}}_{1}\right)=arg\;{max}_{\left(\lambda_{1},w_{1}\right)}\ell \left(\lambda_{1},w_{1};\lambda_{2},\overset{ˆ}{f},{𝒲}_{N_{k}}\right).$$

The notation used here follows the conventions established in Huang et al. [23].

- *k* = 1, … , *N_k_* is the index of the PLI events.
- *N_k_* is the total number of PLI events acquired.
- *c_k_* is the PET-TOF LOS in which the *k*th PLI event is detected.
- *τ_k_* is the measured elapse time of the *k*th PLI event.
- ${𝒲}_{N_{k}}=\left\{\left(c_{k},\tau_{k}\right):1\leq k\leq N_{k}\right\}$ is the set of acquired PLI events.
- *λ*_1,_*_j_* is the rate constant of the o-Ps population in the *j*th pixel. ***λ*_1_** is called o-Ps rate-constant image.
- *j* = 1, … , *N_j_* is the index of the pixel.
- *N_j_* is the total number of image pixels.
- *λ*_2,_*_j_* is the rate constant of the DA population in the *j*th pixel. ***λ*_2_** is called the DA rate-constant image.
- *w*_1,_*_j_* is the population weight of o-Ps in the *j*th pixel. ***w*_1_** is called the o-Ps weight image.
- *w*_2,_*_j_* is the population weight of DA positrons in the *j*th pixel. ***w*_2_** is called the DA weight image.
- *f_j_* is the number of decays in the *j*th pixel. ***f*** is the activity image.
- *σ* is Gaussian error of the EMG distribution, which is set to value $\sqrt{3/2}\sigma_{t}$, as shown in [24].
- *H_c,j_* is an element of the system matrix ***H*** that is proportional to the probability that an isotope decay occurring inside pixel *j* would give rise to a two-photon coincidence event at LOS *c*.

*w*_1,_*_j_* and *w*_2,_*_j_* in Eq. (1) and Eq. (2) are the proportions of PLI events originating in pixel *j* that come from the o-Ps and DA populations, respectively. Evidently, $w_{1,j}+w_{2,j}=1$, yielding $w_{2}=1-w_{1}$ in Eq. (3).

In this paper, we assume that the DA rate-constant image ***λ*_2_** is known (***λ*_2_** = 2.5 ns^−1^) and focus on the reconstruction of the o-Ps rate-constant image. However, the proposed method can be readily extended to the scenario where the DA and o-Ps rate-constant images are both unknown. This will be investigated in future work.

### 2.3 Maximum likelihood estimation and performance evaluation

The MLE of ***f***, denoted as $\overset{ˆ}{f}$, was obtained using 50 iterations of the expectation–maximization algorithm, Lange and Carson [27], implemented in-house in Python. A total of 50 iterations were empirically determined as sufficient to reach convergence. No explicit noise regularization schemes were employed. The MLE of ***λ*_1_**, denoted as $\overset{ˆ}{\lambda }$, and its weight ***w*** was obtained by solving Eq. (4) using the limited-memory Broyden–Fletcher–Goldfarb–Shanno bound (L-BFGS-B) algorithm [28] that is available from scipy.optimize in Python, due to its ability to deal with large numbers of variables and for imposing the positivity condition for the solution (implemented using the bound constraints).

To evaluate the reconstruction performance, for each simulation, we calculated the normalized mean square error (NMSE) and the contrast recovery coefficient (CRC, NEMA Standards Publication NU2-2001 [29]). NMSE measures the mean squared error of the estimated image in comparison with the true image, normalized by the squared Euclidean norm of the true image. CRC measures the degree of recovery of the true contrast of two circular regions compared to the background. Lower values of NMSE and higher values of CRC are indicative of better performance. Additionally, we proposed using a novel performance metric the standardized absolute log ratio (SALR) that is defined as

(5) $${SALR}_{p}=\frac{\left|log\left(\frac{{\bar{\overset{ˆ}{\lambda }}}_{1,p}}{{\bar{\overset{ˆ}{\lambda }}}_{b}}\right)\right|}{SD\left({\overset{ˆ}{\lambda }}_{b}\right)/{\bar{\overset{ˆ}{\lambda }}}_{b}},\;p=1,2,$$

where SD stands for standard deviation, $\bar{\cdot }$ means the average, *p* = 1, 2 represents the left and right circle in the phantom, and the subscript *b* represents the background in the phantom. In Eq. (5), the denominator represents the background variability of the reconstructed image due to noise, as described in Raczyński et al. [30]. The SALR assesses the contrast of two circular regions relative to background variability. We calculate an overall SALR by averaging SALR_1_ and SALR_2_. Unlike other metrics, the SALR does not require knowledge of the ground-truth image. Similar to CRC, higher SALR values indicate better performance. Additional details on the computation of NMSE and CRC can be found in the Supplementary Material.

## 3 Results

### 3.1 Two-component decay model outperformed the single-component decay model

We first implemented the two-component model with the assumption that ***w*_1_** is known and used its true value. Thus, the only unknown variable was the o-Ps rate-constant image. The true ***w*_1_** = 0.3, ***w*_2_** = 0.7, and ***λ***_2_ = 2.5 ns^−1^ were used for obtaining the MLE of ***λ***_1_. In addition, the estimated activity image $\overset{ˆ}{f}$ was used.

**FIGURE 3:**
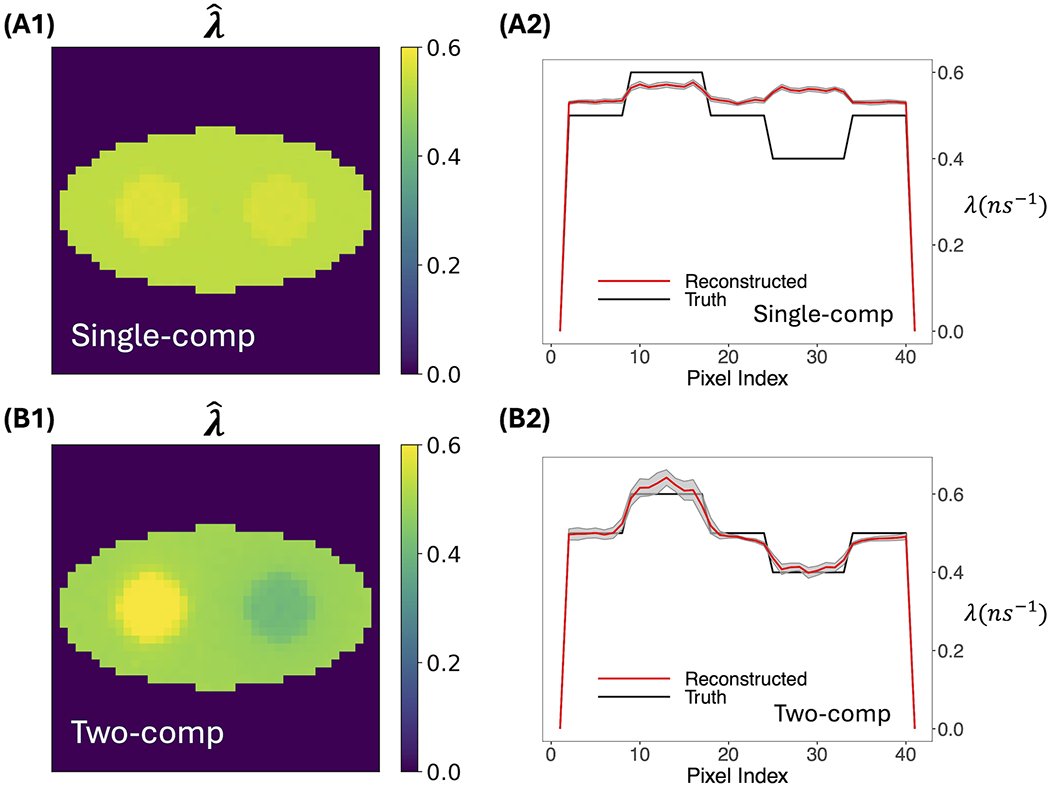
Average of 20 reconstructed o-Ps rate-constant images and their horizontal profiles across the center of image obtained from 20 simulation runs. The gray shaded areas in the horizontal profile plots are the ±1 SD range computed from the 20 images. **(A1, A2)** are results obtained by the single-component model. **(B1, B2)** are results obtained by the two-component model. For the two-component model, the true ***w*_1_**, ***w*_2_**, and ***λ***_2_ were used. For *Λ* both models, estimated activity image $\overset{ˆ}{f}$ was used. Note that, as described in the text, data were simulated using three positron populations.

Figure 3 compares o-Ps rate-constant images obtained using the two-component model *versus* using the previous single-component model. Figure 3A1, A2 show the averaged reconstructed image from 20 simulation runs (called mean images below). Figure 3B1, B2 plot the pixel values across the center row of the true and average reconstructed image (called horizontal profiles below). The gray shaded regions in these horizontal profiles represent the ±1 SD range of the estimated rate-constant image, calculated from the 20 simulation runs. Visually, the mean image of the two-component model agrees with the ground truth, while that of the single-component model barely resolves the two circles in the phantom. The horizontal profile of the two-component model also quantitatively agrees with the ground truth, while the profile of the single-component model is almost flat and shows substantial bias in the circles and in the background. The deviation is particularly pronounced for the right circle, which has a rate constant of 0.4 ns^−1^. We believe that the result of the single-component model is biased toward the DA population that is not accounted for. This DA population has a large rate constant of 2.5 ns^−1^; therefore, it generally leads to a positive bias in the reconstructed o-Ps rate-constant image. Compared to the single-component model, the two-component model yields greater SDs. However, they are small relative to the contrast of the circles, indicating stability of reconstruction.

**FIGURE 4:**
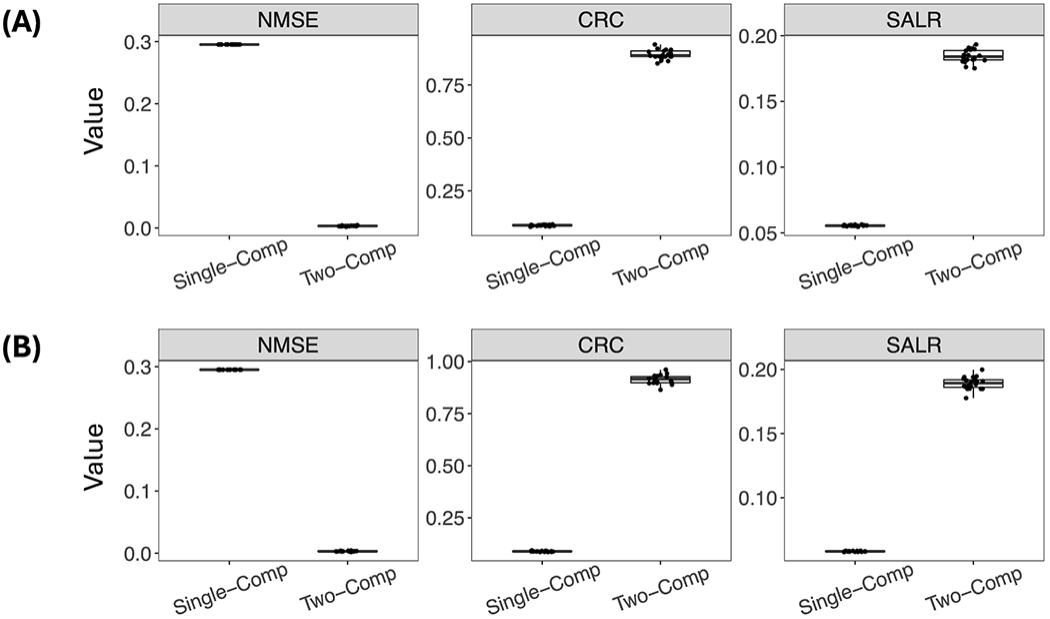
Performance of reconstruction using the single-component model and the two-component model evaluated by three performance metrics. For the two-component model, the true **w_1_**, **w_2_**, and **λ**_2_ were used. **(A)** Using the estimated $\overset{ˆ}{f}$. **(B)** Using the true ***f***.

Figure 4 further shows box plots of the three performance metrics–NMSE, CRC, and SALR, obtained from 20 reconstructed images using the single-component and two-component models. In addition to using the true activity image ***f***, we also used the estimated activity image $\overset{ˆ}{f}$ obtained using the OSEM algorithm for the realistic scenario in which the true ***f*** is unavailable. The two-component model demonstrated significantly superior performance in terms of all three metrics. On the other hand, no significant difference in these performance metrics was observed between using the estimated $\overset{ˆ}{f}$ (top row) and true ***f*** (bottom row).

### 3.2 Two-component decay model when weights are unknown

Results shown above were obtained by assuming that the o-Ps weight image ***w***_1_ is exactly known. We relaxed this assumption to allow unknown ***w***_1_ to be estimated together with ***λ*_1_** by MLE. However, we assumed a uniform weight image ***w***_1_ whose shape is known (the background ellipse), but the value is not. We believe that this is a reasonable assumption because the external boundaries of the rate-constant and weight images can be readily determined from the activity image.

We extended our investigation to evaluate the performance of the two-component model across various PLI event sizes and TOF resolutions. The previous study by Huang et al. [23] used 1 million simulated PLI events, which is equivalent to over 1,500 events per pixel. We conducted experiments with event sizes ranging from 100 to 1,000 events per pixel to evaluate the practicality and efficiency of the proposed model. Additionally, we examined the effect of TOF resolution on model performance.

**FIGURE 5:**
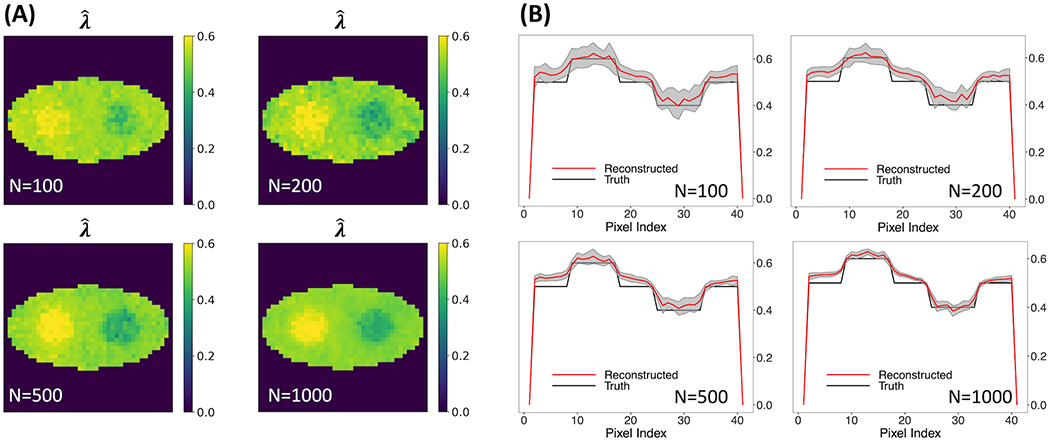
Reconstructed rate-constant images and horizontal profiles across the center of the reconstructed images for various numbers of events per pixel.**(A)** Rate-constant images from a single simulation run. **(B)** Horizontal profiles of true and reconstructed rate-constant image.

Figure 5A shows the rate-constant images of a single simulation run obtained using the two-component model, allowing unknown o-Ps population weight. Various numbers of events per pixel, ranging from 100, 200, 500 to 1,000, were examined. The images in Figure 5A show stability when the number of events per pixel is decreased from 1,000 to 100. In all cases, the two circles can be readily identified.

Figure 5B shows the horizontal profile of the mean images, along with the ±1 SD range, for the four event sizes examined. The horizontal profile for 100-event-per-pixel case agrees with the truth, with a larger SD in comparison with the 1000-event-per-pixel case. Compared to Figure 3 (B2) that is obtained using true ***w*_1_**, a small positive bias can be observed. These results demonstrated that the two-component method has potential to produce quantitatively an accurate o-Ps rate-constant image with a limited number of events.

**FIGURE 6:**
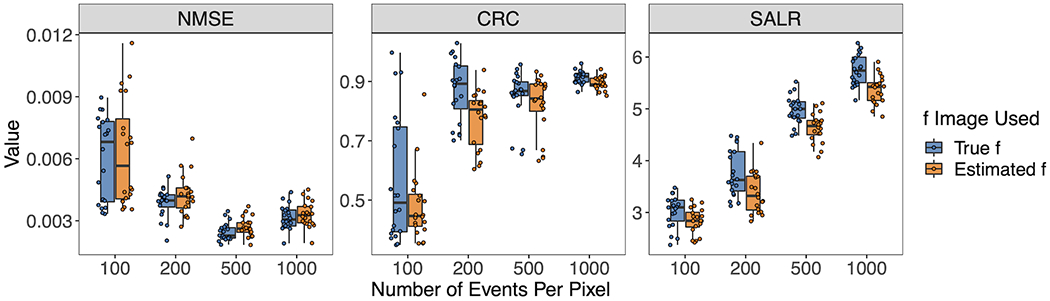
Box plots of NMSE, CRC, and SALR for various numbers of events per pixel, using true and estimated ***f***.

Figure 6 shows the three performance metrics when the number of pixels per pixel varies. Again, the true ***f*** or estimated $\overset{ˆ}{f}$ was used. Generally, reduction in the event size degrade the reconstruction performance and introduces greater variability for all three metrics. However, the degradation in the performance metrics appears mild, except for the 100-event-per-pixel case. Notably, unlike NMSR and CRC, the SALR demonstrates a clear monotonically increasing trend as the number of events per pixel increases. In addition, it has a very constant range of variability. Such results highlight the interpretability of SALR for evaluation of image quality and image reconstruction methods.

In addition to evaluating the performance using the global CRC, SALR, and NMSE calculated from the entire reconstructed images, we also calculated the SD and bias of the three regions of interest in the reconstructed image (left circle, background, and right circle) to assess the local performance properties. The results are shown in Supplementary Figure S1 in Supplementary Material. In general, the SD decreases with the reduction of the event sizes, and bias does not decrease drastically, except for the event per pixel of 100. Details of the calculation of the bias and SD are provided in the Supplementary Material.

**FIGURE 7:**
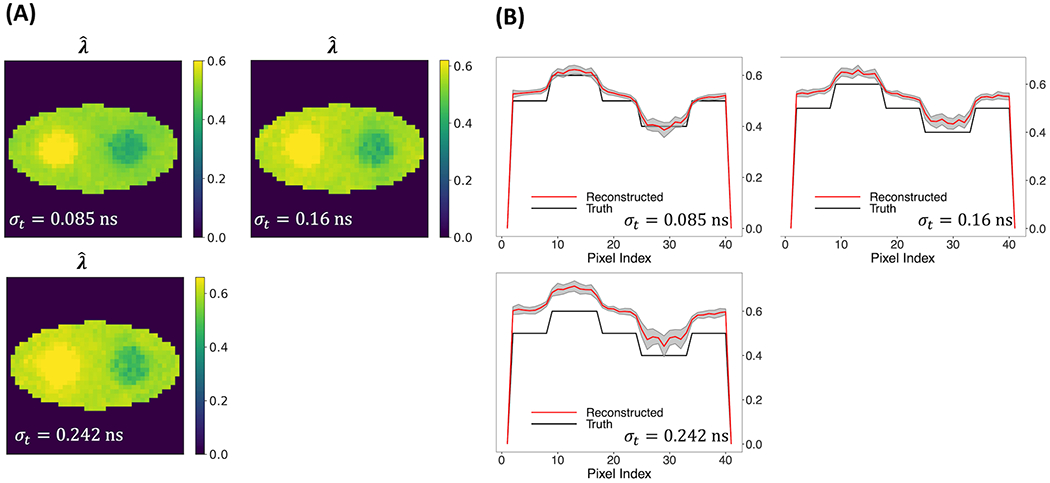
Reconstructed rate-constant images and horizontal profiles across the center of the reconstructed images for various TOF resolutions. **(A)** Rate-constant images from a single simulation run. *(B)* Horizontal profiles of true and reconstructed rate-constant image.

Furthermore, we examined three TOF resolutions, i.e., *σ_t_* = 0.085 ns, 0.16 ns, and 0.242 ns, investigating how the reconstruction performance is affected. Figure 7A shows that the new two-component model captures the two circles even when *σ_t_* is as large as 0.242 ns (570 ps CRT). An increasing positive bias of the reconstructed $\overset{ˆ}{\lambda }$ can be observed in Figure 7 as TOF resolution increases, which indicates that under higher TOF resolution, the reconstructed $\overset{ˆ}{\lambda }$ is more affected by the slow-decay component, which has a higher decay-rate constant. Figures 7A, B also show slightly increasing noise and variability in $\overset{ˆ}{\lambda }$ with increasing TOF resolution.

**FIGURE 8:**
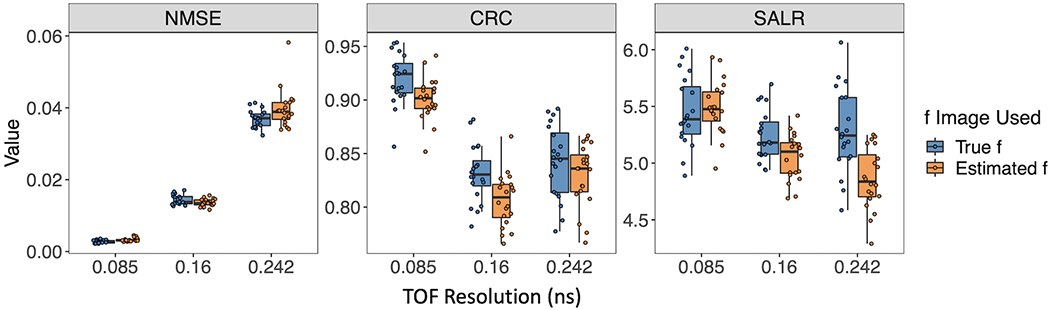
Box plots of CRC, NMSE, and SALR for various TOF resolutions, using true and estimated ***f***.

In general, Figure 8 demonstrates a decline in the performance of three metrics, with the exception of an improvement in CRC, as TOF resolution increases from 0.16 ns to 0.242 ns. This can be explained by the fact that CRC evaluates the degree of recovery in the comparison between two circular regions and the background of the reconstructed image. A higher CRC does not necessarily indicate a higher-quality image, as it does not account for the quantitative accuracy and variability of the reconstructed image.

### 3.3 Two-component decay model when weights are unknown and non-uniform

Last, we allowed non-uniform weight image ***w***_1_ but with the known shape. This assumption greatly increased the number of variables and introduced great complexity in estimation. Therefore, a larger event size of 2,000 events per pixel was used. Again, 20 simulation runs were used to compute the mean and SD images.

**FIGURE 9:**
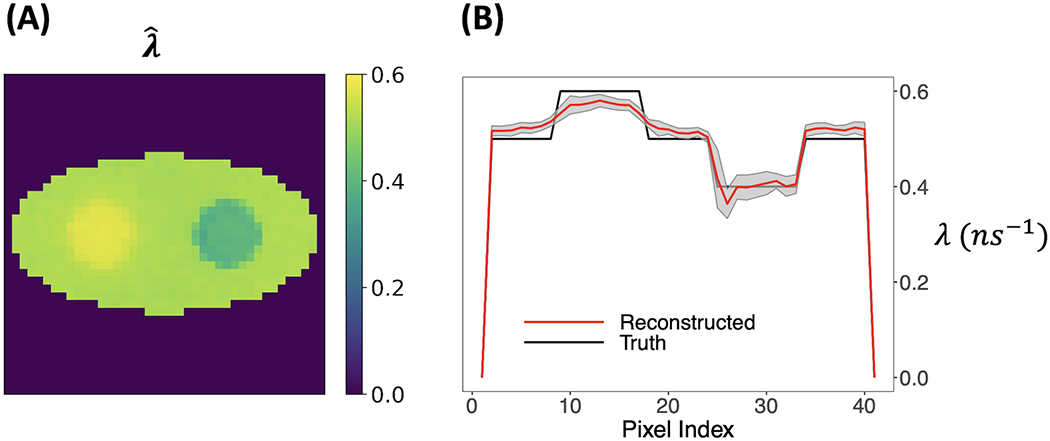
Rate-constant image obtained by the two-component model, allowing a non-uniform o-Ps weight image. **(A)** Mean image from 20 simulation runs. **(B)** Horizontal profile across the center of the image and the ±1 SD range.

Figure 9A shows that the mean o-Ps rate-constant image maintained a high contrast of the two circles. The horizontal profile in Figure 9B again agreed well with the ground truth, and the ±1 SD range is reasonably small relative to the contrast of the circles.

**FIGURE 10:**
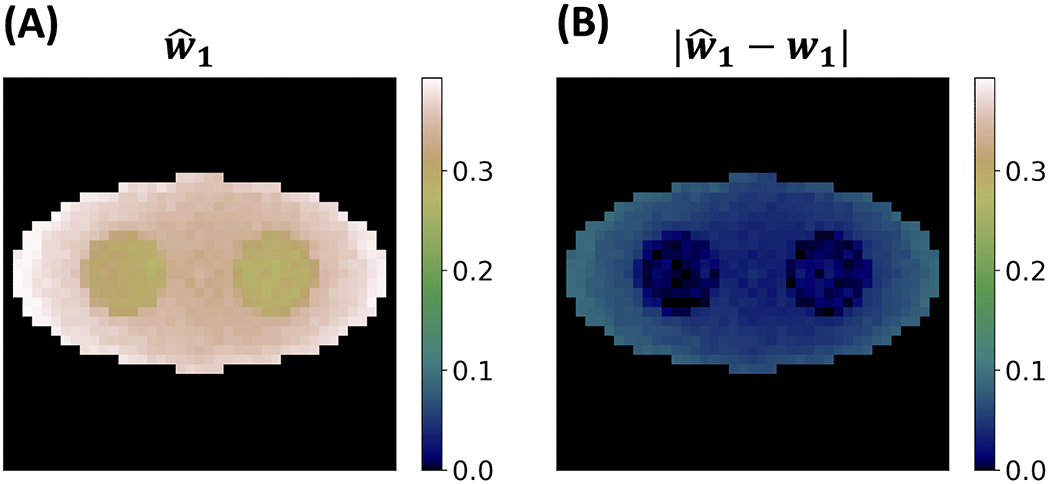
Weight reconstruction results for o-Ps using the two-component model from a single simulation run. **(A)** Reconstructed weight image. **(B)** Absolute value of the difference between the reconstructed weight image and the ground truth.

Figure 10 shows the estimated weight image ${\overset{ˆ}{w}}_{1}$ and its absolute difference with the ground truth ***w*_1_**. Figure 10A illustrates a high degree of accuracy of the estimated weight image as it closely approximates the true value of 0.3 across the elliptical background. The absolute difference image in Figure 10B further elucidates this precision. The results in Figures 9, 10 highlight that despite the greatly increased number of variables in estimation, the proposed two-component model with ML approach can still yield reliable solutions.

## 4 Discussion

This study presents a significant advancement in PLI through the development of a two-component reconstruction model in TOF-PET. By incorporating both a slow-decay and short-decay component, our model offers a more accurate description of the PLI data. The simulation studies conducted in this research clearly demonstrate the superior performance of the two-component model in capturing the intricacies of the o-Ps rate-constant image compared to traditional single-component models. This highlights the potential of our model in enhancing the accuracy of PLI, thereby contributing to more precise and informative medical imaging. The results of this study open up new possibilities for improving disease diagnosis and treatment planning, ultimately leading to better patient outcomes. Our work also lays the groundwork for further research in this area, which could lead to the development of even more advanced imaging techniques and technologies.

In this paper, the PLI data and rate-constant images are restricted to 2D cases. Further investigations are warranted to extend the proposed method to 3D settings. Additionally, the current two-component decay model treats ***λ*_2_**, the slow-decay component, as known. The model can be readily extended to incorporate ***λ*_2_** as an unknown variable by providing its derivative to the optimization algorithm. However, this extension will introduce substantial computational complexities, which will be explored in future work.

Future studies should further investigate the robustness and accuracy of the model under low-dose conditions to ensure its applicability in these imaging scenarios. Additionally, extending the model to include three positron populations would allow for a more detailed characterization of the positronium decay processes for yielding further improvements in PLI. It is also of interest to add regularization to the objective function for estimating the rate-constant image.

## Supplementary Material

supplement

The Supplementary Material for this article can be found online at: https://www.frontiersin.org/articles/10.3389/fphy.2024.1429344/full#supplementary-material

## Data Availability

The raw data supporting the conclusion of this article will be made available by the authors, without undue reservation.
