# Supplementary material for: Enhanced positronium lifetime imaging through two-component reconstruction in time-of-flight positron emission tomography
Source: Front Phys. Author manuscript; Available in PMC 2026 Jan 24. (PMC12829466; doi:10.3389/fphy.2024.1429344)
Supplement: supplement [file NIHMS2133029-supplement-supplement.pdf]

Supplementary Material

1 PERFORMANCE METRICS

To assess the quality of the reconstructed lifetime images, we modified the contrast recovery coefficient (CRC), previously described in Raczyński et al. (2020), to apply to lifetime data instead of count-data.

The CRC for each region of interest is expressed as:

$$\mathrm{CRC}_p = \frac{\overline{\widehat{\lambda}_p}/\overline{\widehat{\lambda}_b} - 1}{\lambda_p/\lambda_b - 1}, \quad p = 1, 2, \tag{S1}$$

where $\overline{\widehat{\lambda}_p}$ denotes the average decay rate over the pixels within disc p of the reconstructed image, $\overline{\widehat{\lambda}_b}$ represents the average decay rate over the background pixels, λ_p is the true decay rate for disc $p, p = 1, 2$ ($\lambda_1 = 0.6$ ns^{-1}, $\lambda_2 = 0.4$ ns^{-1}), and $\lambda_b = 0.5$ ns^{-1} for the background. The overall CRC is the average of CRC_1 and CRC_2.

Additionally, the accuracy of the reconstruction is quantified using the normalized mean square error (NMSE), defined as:

$$\mathrm{NMSE} = \frac{\|\hat{\boldsymbol{\lambda}} - \boldsymbol{\lambda}\|^2}{\|\boldsymbol{\lambda}\|^2}, \tag{S2}$$

where $\hat{\boldsymbol{\lambda}}$ and $\boldsymbol{\lambda}$ correspond to the reconstructed and true decay-rates in the images, respectively, and $\|\cdot\|$ indicates the L-2 norm.

Moreover, as detailed in the manuscript, we introduce a new metric, the standardized absolute log ratio (SALR), which measures the contrast between regions of interest and the background while adjusting for the background variability of the reconstructed images:

$$\mathrm{SALR}_p = \frac{\left|\log\left(\frac{\bar{\hat{\boldsymbol{\lambda}}}_{1,p}}{\bar{\hat{\boldsymbol{\lambda}}}_b}\right)\right|}{\mathrm{SD}\left(\hat{\boldsymbol{\lambda}}_b\right)/\bar{\hat{\boldsymbol{\lambda}}}_b}, p = 1, 2 \tag{S3}$$

where $p = 1, 2$ denotes the left and right discs in the phantom, and the subscript b is used for the background. The average of SALR_1 and SALR_2 is used to evaluate this metric.

In addition to performance metrics calculated for the entire reconstructed decay-rate image, we analyze the bias and standard deviation (SD) of the proposed method for three specific regions within the image: the left circle with a decay rate of $\lambda = 0.6$ ns^{-1}, the background with a decay rate of $\lambda = 0.5$ ns^{-1}, and the right circle with a decay rate of $\lambda = 0.4$ ns^{-1}. To compute the bias for each region, we first calculate the absolute value of the element-wise difference between the averaged reconstructed decay-rate image and the ground-truth image, as

$$\left|\frac{1}{N_{\text{sim}}}\sum_{i=1}^{N_{\text{sim}}}\hat{\boldsymbol{\lambda}}_i - \boldsymbol{\lambda}\right|, \tag{S4}$$

where $N_{\text{sim}} = 20$ is the number of independent runs for each simulation setting. We then average these bias values within each region to obtain a final bias value.

Similarly, to calculate the SD, we first compute the element-wise standard deviation across the 20 reconstructed decay-rate images to obtain a matrix of SD values. We then average these SD values within each region to obtain a final SD value.

2 SUPPLEMENTARY TABLES AND FIGURES

2.1 Figures

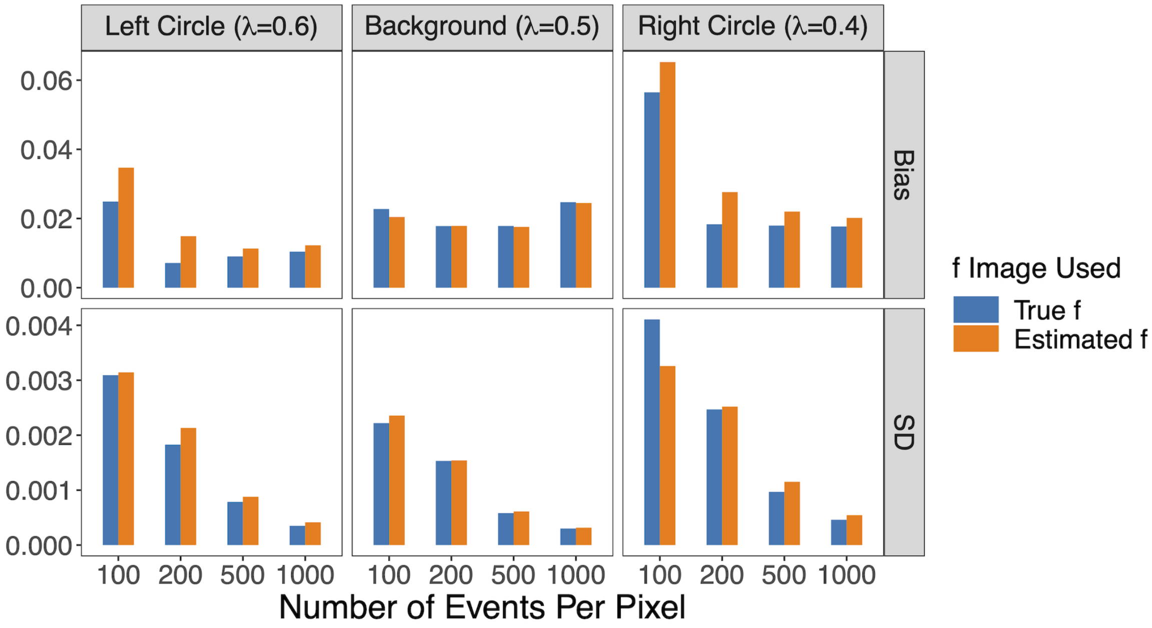

Figure S1. Bias and standard deviations of the ML estimation for the three regions (left circle, background, right circle) of interest, using various numbers of events per pixel, and using true and estimated $\boldsymbol{f}$.

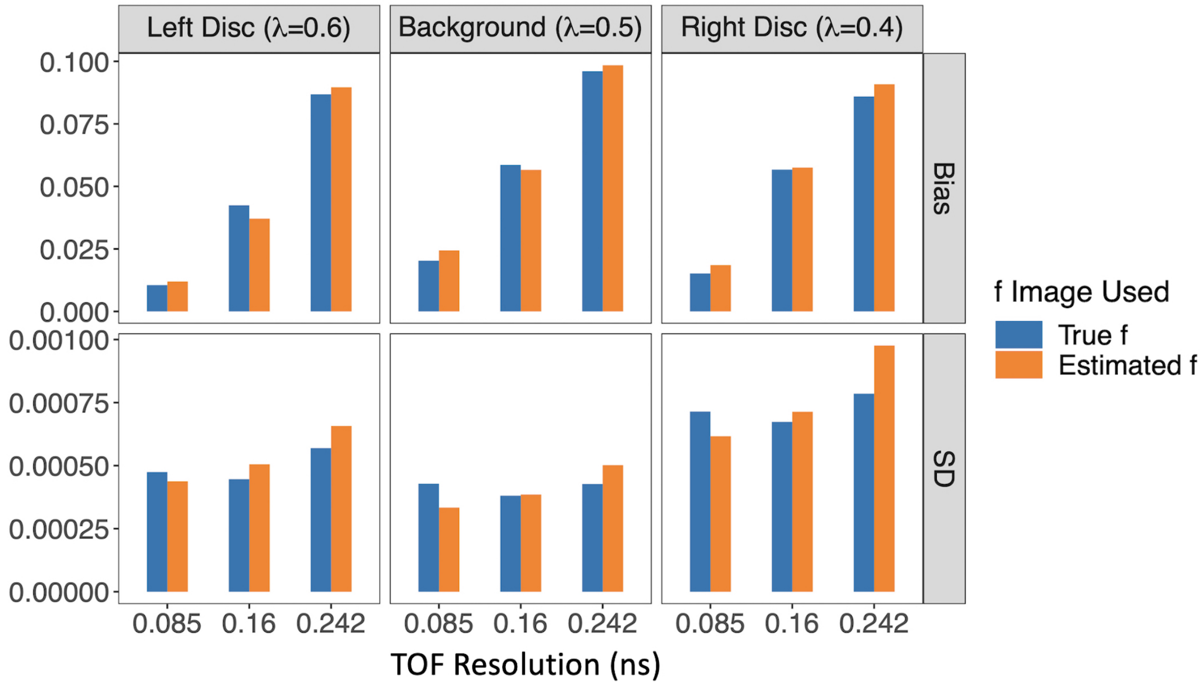

Figure S2. Bias and standard deviations of the ML estimation for the three regions (left circle, background, right circle) of interest, using various TOF resolutions, and using true and estimated $\boldsymbol{f}$.

REFERENCES

Raczyński, L., Wiślicki, W., Klimaszewski, K., Krzemień, W., Kopka, P., Kowalski, P., et al. (2020). 3D TOF-PET image reconstruction using total variation regularization. *Physica Medica* 80, 230–242
